# Supplementary material for: Every-other-day feeding extends lifespan but fails to delay many symptoms of aging in mice
Source: Nat Commun. 2017 Jul 24;8:155. doi: 10.1038/s41467-017-00178-3 (PMC5537224; doi:10.1038/s41467-017-00178-3)
Supplement: Supplementary file 2 — Supplementary Information [file 41467_2017_178_MOESM2_ESM.pdf]

File Name: Peer Review File
Description:

File Name: Supplementary Information
Description: Supplementary Figures, Supplementary Tables.

File Name: Supplementary Data 1
Description: List of differentially expressed genes in the brain of AL or EOD mice

File Name: Supplementary Data 2
Description: Analyses of differentially expressed genes in the brain of AL or EOD mice using Ingenuity Pathway Analysis software

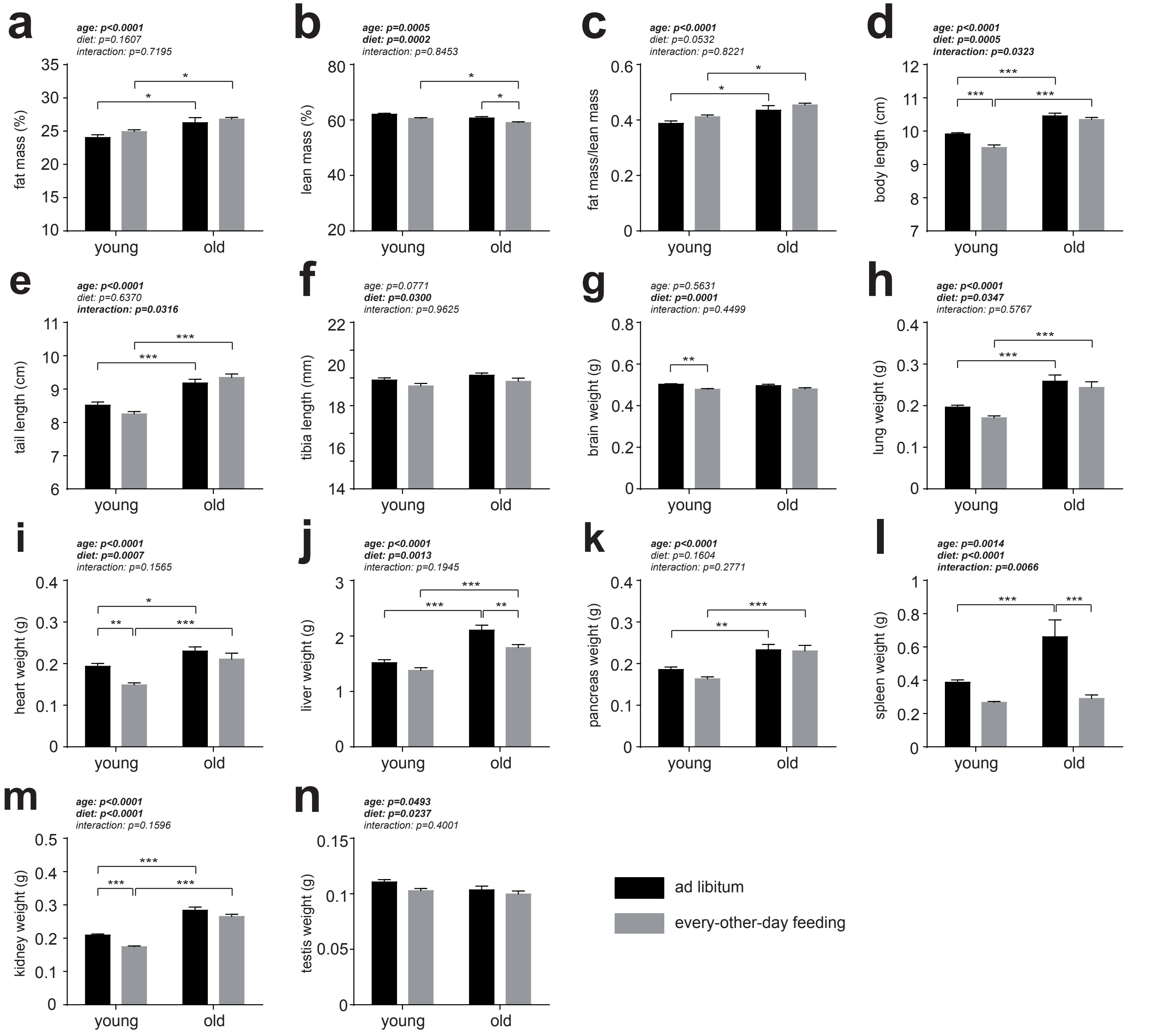

Supplementary Figure 1

Body composition and organ weights. (**a**) Fat mass, (**b**) lean mass, and (**c**) fat/lean mass ratio were determined using a body composition analyzer (2-way ANOVA; young+AL: n = 16, young+EOD: n = 16, old+AL: n = 20, old+EOD: n = 22). (**d**) Body length, (**e**) tail length, (**f**) tibia length and weights of (**g**) brain, (**h**) lung, (**i**) heart, (**j**) liver, (**k**) pancreas, (**l**) spleen, (**m**) kidney, and (**n**) testis as measured in young and aged mice (2-way ANOVA; young+AL: n = 15, young+EOD: n = 15, old+AL: n = 11, old+EOD: n = 10). AL = ad libitum, EOD = every-other-day feeding. Data are presented as mean ± SEM.

Main leukocyte populations

Leukocytes
(CD45$^+$)

B cell definition:
CD19$^+$ B220$^+$

Non-B cells
(CD19$^-$ B220$^-$)

T cell definition:
CD3$^+$ CD5$^+$

Non-B non-T cells
(CD19$^-$ B220$^-$/CD3$^-$ CD5$^-$)

Granulocyte definition:
Gr-1$^+$ CD11b$^+$

Non-B non-T cells non-granulocyte
(CD19$^-$ B220$^-$/CD3$^-$ CD5$^-$/Gr-1$^-$)

NK cell definition:
NKp46$^+$ or NK1.1$^+$

Non-B non-T cells
non-granulocyte non-NK cells
(CD19$^-$ B220$^-$/CD3$^-$ CD5$^-$/Gr-1$^-$/NKp46$^-$ NK1.1$^-$)

Monocyte definition:
CD11b$^+$

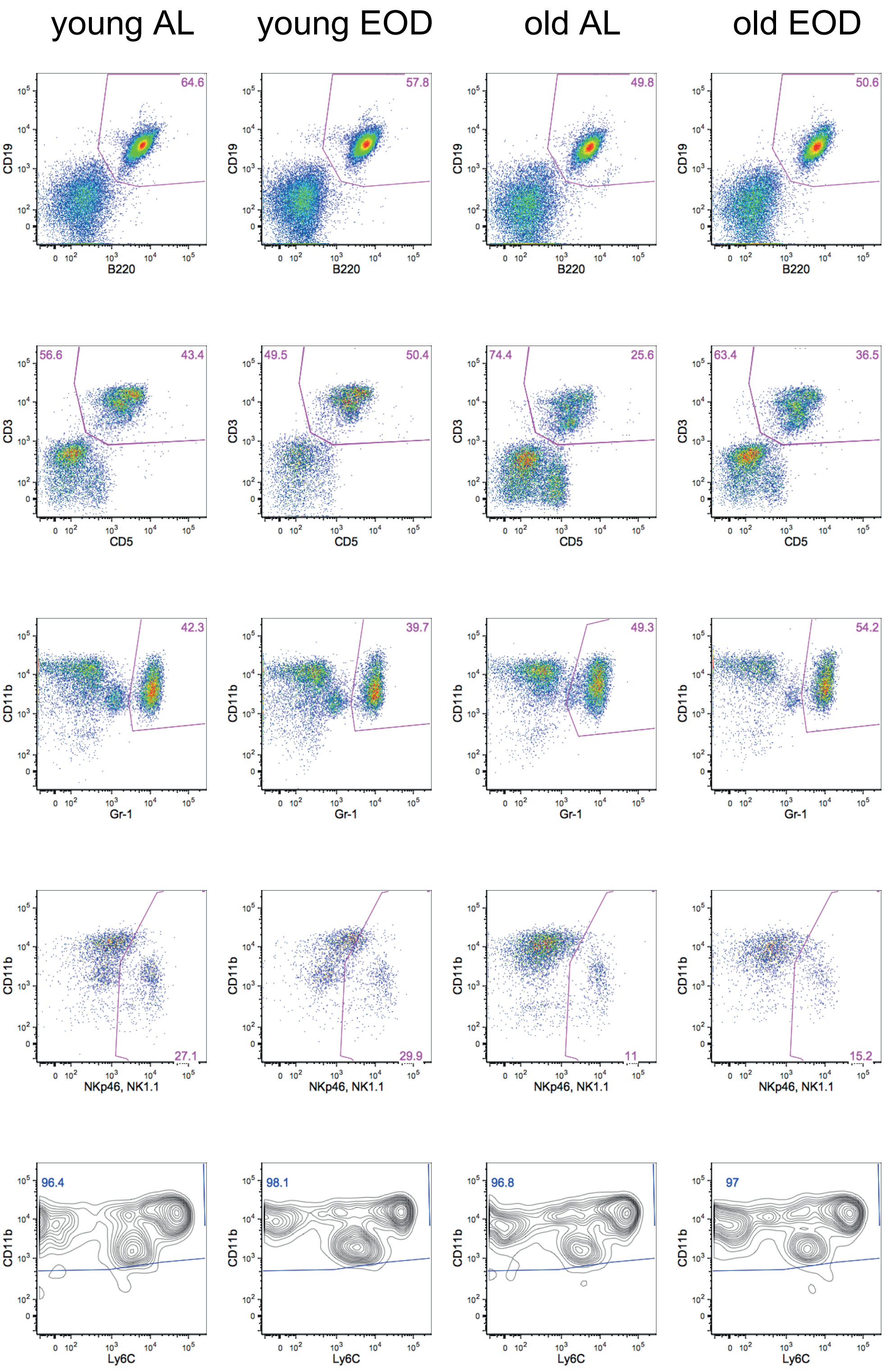

Numbers within the gates indicate percentages of the parent gate

Supplementary Figure 2
Definition and gating strategy of main peripheral blood leukocytes analyzed using a 10-color flow cytometer.

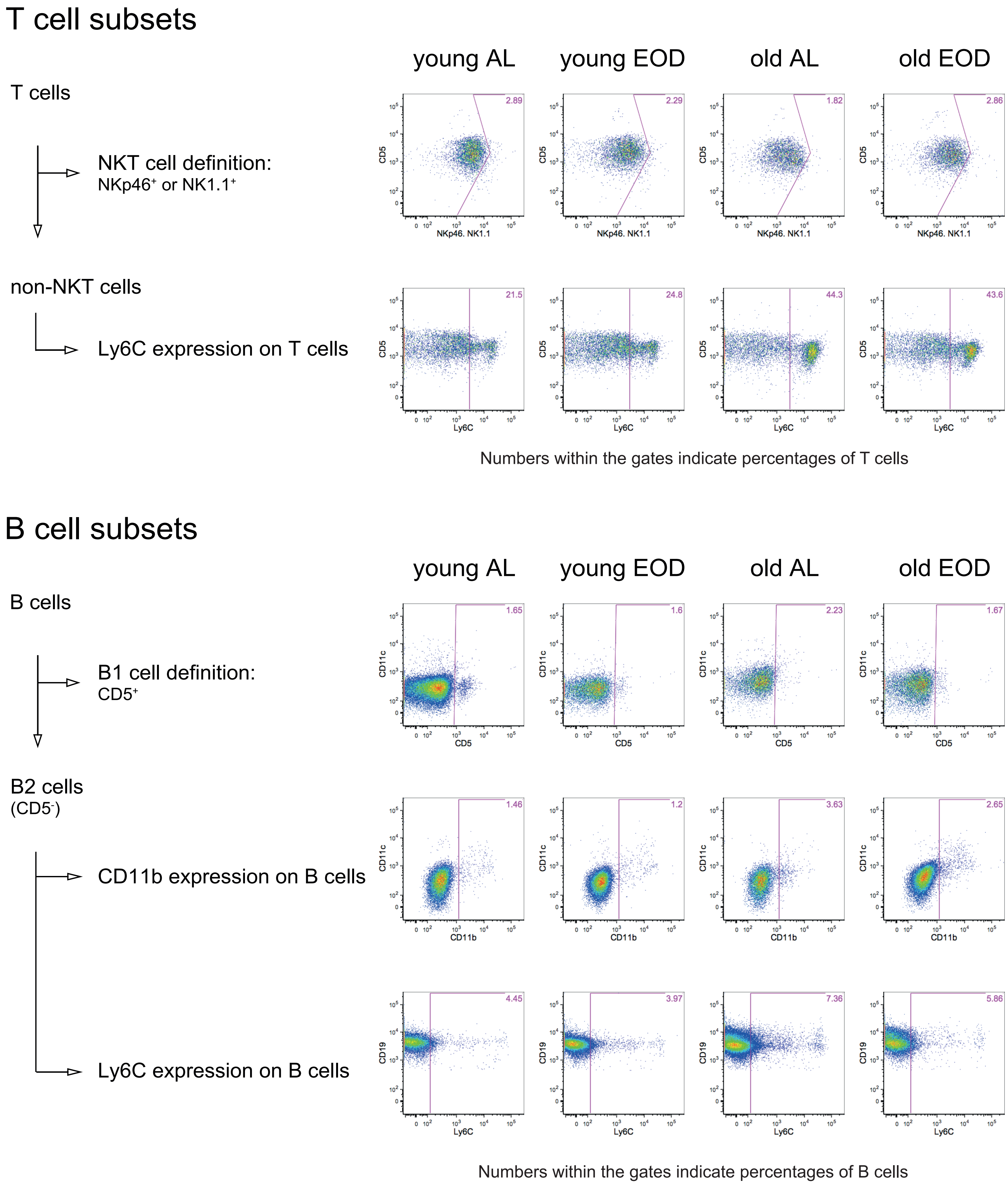

Supplementary Figure 3
Definition and gating strategy of T and B cell subsets analyzed using a 10-color flow cytometer.

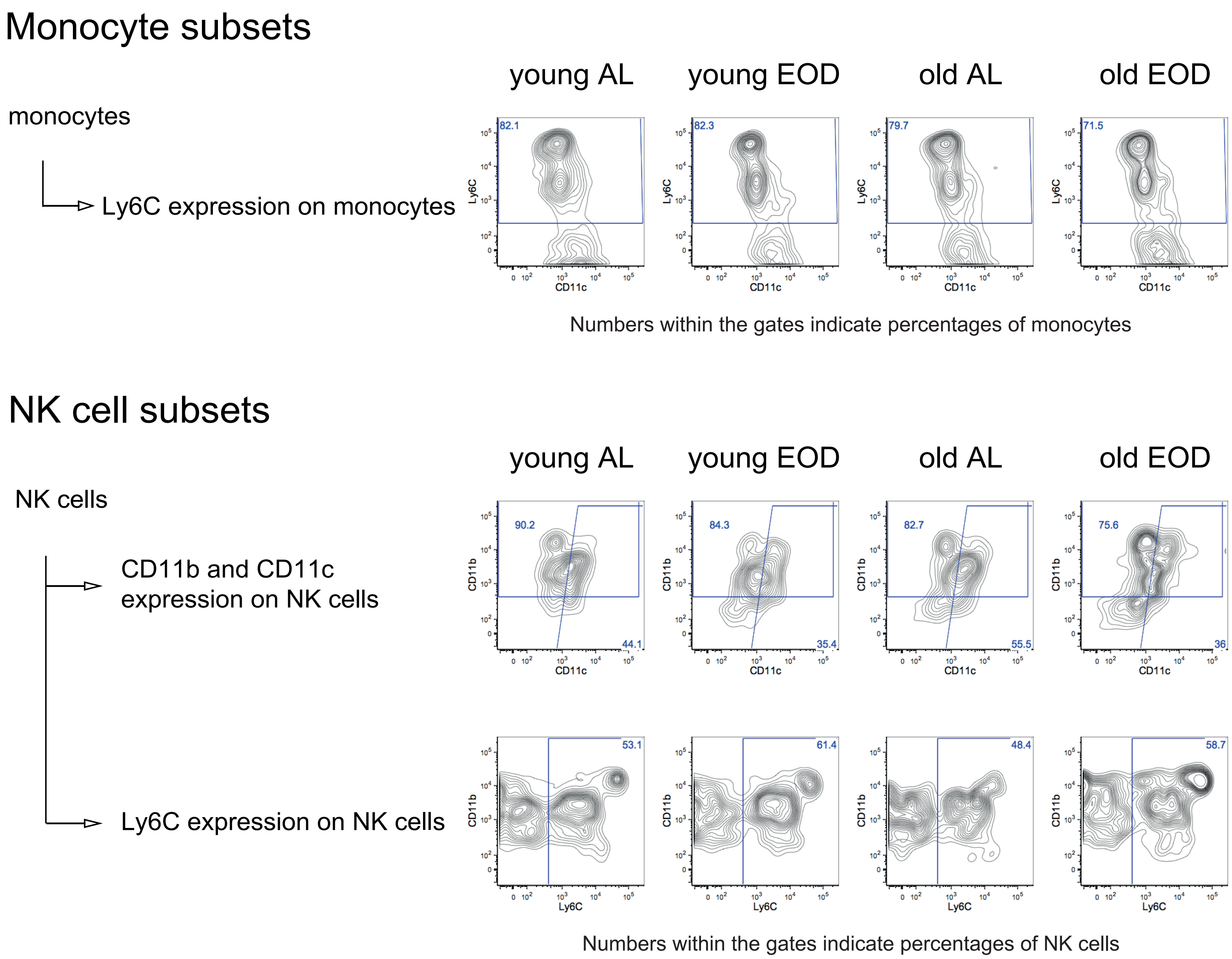

Supplementary Figure 4
Definition and gating strategy of monocyte and NK cell subsets analyzed using a 10-color flow cytometer.

Supplementary Table 1

Tumor burden in mice that died of natural causes and were kept on either lifelong ad libitum access to food or on every-other-day feeding. The numbers of tumors per animal are shown as mean +/- SEM and differed significantly between groups (t-test, *p*=0.0308).

	ad libitum (n = 29)	every-other-day feeding (n = 20)	p-value
**number of malignant tumors**	**31 (= 83.8% of tumors)**	**16 (= 94.1% of tumors)**	
lymphoma	13	6	
bronchoalveolar carcinoma	7	1	
histiocytic sarcoma	5	3	
hepatocellular carcinoma	3	4	
leukemia	1	0	
papillary colon carcinoma	1	1	
fibrosarcoma	1	0	
**number of benign tumors**	**6 (= 16.2% of tumors)**	**1 (= 5.9% of tumors)**	
papillary thyroid adenoma	4	0	
bronchoalveolar adenoma	2	1	
**overall tumor burden**	**37 tumors in 29 animals**	**17 tumors in 20 animals**	
**tumor per animal**	**1.29 ± 0.13**	**0.85 ± 0.13**	**0.0308**

Supplementary Table 2

Aging phenotypes prevented by every-other-day feeding. Aging phenotypes attenuated by every-other-day feeding specifically in old mice on lifelong treatment without apparent changes in young animals on short-term treatment.

Test	Parameter	Aging phenotype	Effect of every-other-day feeding Aged mice/ lifelong EOD	Young mice/ short-term EOD
Brain				
Histopathology	Eosinophilic thalamic inclusions	Increased	Decreased	Not applicable
Clinical chemistry				
Clinical chemistry	Plasma urea concentration	Increased	Decreased	No effect
Clinical chemistry	Plasma insulin concentrating after fasting	Increased	Decreased	No effect
Immune system				
Immunology	NKT cell frequencies	Decreased	Increased	No effect
Kidney				
Histopathology	Urinary casts	Increased	Decreased	No effect
Liver				
Histopathology	Microgranuloma	Increased	Decreased	No effect
Metabolism				
Indirect calorimetry	Respiratory exchange rate	Decreased	Increased	No effect

Supplementary Table 3

Age-sensitive traits ameliorated by every-other-day feeding in aged mice on lifelong treatment and with similar effects in young mice on short-term treatment.

Test	Parameter	Aging phenotype	Effect of every-other-day feeding: Aged mice/ lifelong EOD	Young mice/ short-term EOD
Behavior and cognition				
Open field	Exploratory activity-related measures	Decreased	Increased	Increased
Open field	Resting duration	Decreased	Increased	Increased
SHIRPA	Locomotor activity	Decreased	Increased	Increased
Body size and organ weights				
Body size	Body length	Increased	Decreased	Decreased
Organ weight	Lung weight	Increased	Decreased	Decreased
Organ weight	Heart weight	Increased	Decreased	Decreased
Organ weight	Liver weight	Increased	Decreased	Decreased
Organ weight	Kidney weight	Increased	Decreased	Decreased
Organ weight	Spleen weight	Increased	Decreased	Decreased
Clinical chemistry				
Clinical chemistry	Plasma lactate dehydrogenase activity	Increased	Decreased	Decreased
Clinical chemistry	Plasma unsaturated iron binding capacity	Increased	Decreased	Decreased
Cardiovascular system				
Echocardiography	Interventricular septum in diastole	Increased	Decreased	Decreased
Echocardiography	Left ventricular inner dimension in diastole	Increased	Decreased	Decreased
Echocardiography	Left ventricular mass	Increased	Decreased	Decreased
Eye				
Virtual drum	Visual acuity	Decreased	Increased	Increased
Hematology				
Hematology	Hemoglobin	Decreased	Increased	Increased
Hematology	Hematocrit	Decreased	Increased	Increased
Hematology	Red blood cell distribution width	Increased	Decreased	Decreased
Hematology	Platelets volume	Increased	Decreased	Decreased
Hematology	Platelets distribution width	Increased	Decreased	Decreased
Immune system				
Immunology	T cell frequencies	Decreased	Increased	Increased
Immunology	$CD11c^+$ NK cell frequencies	Increased	Decreased	Decreased
Kidney				
Histopathology	Tubular luminal space	Increased	Decreased	Decreased
Metabolism				
Indirect calorimetry	Oxygen consumption/body weight	Decreased	Increased	Increased
Indirect calorimetry	Carbon dioxide production/body weight	Decreased	Increased	Increased
Indirect calorimetry	Heat production/body weight	Decreased	Increased	Increased
Indirect calorimetry	Water consumption/body weight	Decreased	Increased	Increased
Thyroid gland				
Histopathology	Thyroid follicle size	Increased	Decreased	Decreased

Supplementary Table 4

Aging phenotypes not altered or worsened by every-other-day feeding. Age-sensitive traits not modified by every-other-day feeding are denoted in blue. Age-sensitive traits exacerbated by every-other-day feeding are shown in red.

Test	Parameter	Aging phenotype
Behavior and cognition		
Acoustic startle reflex	Startle response measures	Decreased
Auditory brain response	Evoked potentials	Decreased
Hot plate	Latency to respond	Increased
Indirect calorimetry	Activity-related measures (x-axis) in home cage	Increased
Indirect calorimetry	Rearing activity in home cage	Increased
Open field	Latency to enter the center	Increased
Pre-pulse inhibition related measures	PPI measures	Decreased
Rotarod	Motor coordination	Decreased
SHIRPA	Tail elevation	Increased
SHIRPA	Response to acoustic stimulus	Increased
RNA-seq	Gene expression in brain	Aging-associated transcriptional changes
Body composition		
NMR minispec	Fat mass	Increased
NMR minispec	Lean mass	Decreased
NMR minispec	Fat mass/lean mass ratio	Increased
Body size and organ weights		
Body size	Tail length	Increased
Organ weight	Pancreas weight	Increased
Organ weight	Testis weight	Decreased
Bone		
Micro-CT	Marrrow area	Increased
Micro-CT	Mean polar moment of inertia	Increased
Micro-CT	Cortical thickness	Decreased
Cardiovascular system		
Echocardiography	Interventricular septum in systole	Increased
Echocardiography	Left ventricular posterior wall in diastole	Increased
Echocardiography	Stroke volume	Increased
Echocardiography	Cardiac output	Increased
Electrocardiography	Heart rate	Decreased
Electrocardiography	Duration of RR interval	Increased
Electrocardiography	Duration of PQ interval	Decreased
Electrocardiography	Duration of PR interval	Decreased
Electrocardiography	Duration of QRS interval	Increased
Electrocardiography	QTc dispersion	Decreased
Clinical chemistry		
Clinical chemistry	Plasma concentration of non-esterified fatty acids after fasting	Decreased
Clinical chemistry	Plasma sodium concentration	Increased
Clinical chemistry	Plasma potassium concentration	Increased
Clinical chemistry	Plasma alkaline phosphatase activity	Increased

Clinical chemistry	Plasma glucose concentration	Decreased
Clinical chemistry	Plasma albumin concentration	Decreased
Eye		
Scheimpflug imaging	Optical density measures of the lens	Decreased
Hematology		
Hematology	Red blood cell count	Decreased
Hematology	Platelet count	Increased
Hematology	Mean corpuscular hemoglobin concentration	Decreased
Hematology	Platelet large cell ratio	Increased
Hematology	Plateletcrit	Increased
Hematology	OD blood	Increased
Immune system		
Immunology	$Ly6C^+$ T cell frequencies	Increased
Immunology	B2 cell frequencies	Decreased
Immunology	$CD11b^+$ B cell frequencies	Increased
Immunology	$Ly6C^+$ B cell frequencies	Increased
Immunology	NK cell frequencies	Decreased
Immunology	$CD11b^+$ NK cell frequencies	Decreased
Immunology	Granulocyte frequencies	Increased
Immunology	$Ly6C^+$ monocyte frequencies	Increased
Immunology	$Ly6C^-$ monocyte frequencies	Increased
Immunology	$CD45^+$ non-specific cell frequencies	Increased
Ig measurement	IgE plasma concentration	Increased
Lung		
Histopathology	Broncheoli-associated lymphoid tissue	Increased
Muscle		
Grip strength	Forelimb muscle strength	Decreased
Grip strength	4-paw muscle strength	Decreased
Testes		
Histopathology	Seminiferous epithelium	Decreased

Supplementary Table 5

Every-other-day feeding effects on age-insensitive parameters. This table summarizes parameters that were modified by every-other-day feeding but not by age of the animal.

Test	Parameter	Effect of every-other-day feeding<br>Aged mice/ lifelong EOD	Young mice/ short-term EOD
Adrenal gland			
Histopathology	Lipofuscin deposition	Decreased	Decreased
Behavior and cognition			
Indirect calorimetry	Activity-related measures (y-axis) in home cage	Increased	Increased
SHIRPA	Immediate movement at transfer arousal	Increased	Increased
SHIRPA	Absent urination	Decreased	Decreased
Bone size and organ weights			
Bone size	Tibia length	Decreased	Decreased
Organ weight	Brain weight	Decreased	Decreased
Cardiovascular system			
Echocardiography	Left ventricular inner dimension in systole	Decreased	Decreased
Echocardiography	Left ventricular posterior wall in systole	Decreased	Decreased
Echocardiography	Fractional shortening	Increased	Increased
Echocardiography	Ejection fraction	Increased	Increased
Clinical chemistry			
Clinical chemistry	Plasma cholesterol concentration after fasting	Decreased	Decreased
Clinical chemistry	Plasma triglyceride concentration after fasting	Decreased	Decreased
Clinical chemistry	Plasma glucose concentration after fasting	Decreased	Decreased
Clinical chemistry	Plasma HDL concentration after fasting	Decreased	Decreased
Clinical chemistry	Plasma calcium concentration	Decreased	No effect
Clinical chemistry	Plsama phosphate concentration	Decreased	Decreased
Clinical chemistry	Total protein concentration in plasma	Decreased	Decreased
Clinical chemistry	Plasma triglyceride concentration	Decreased	Decreased
Clinical chemistry	Plasma iron concentration	Increased	Increased
Hematology			
Hematology	White blood cell count	Decreased	Decreased
Hematology	Mean corpuscular volume	Increased	Increased
Hematology	Mean corpuscular hemoglobin content	Increased	Increased
Immune system			
Immunology	$CD45^+$ leukocyte counts	Decreased	Decreased
Immunology	$Ly6C^+$ NK cells	Increased	Increased
Kidney			
Histopathology	Vascular hyaline deposition	Decreased	Decreased
Metabolism			
Glucose tolerance	Glucose tolerance	Decreased	Decreased
Skin			
Transepidermal water loss	Transpiration	Increased	Increased

Supplementary Table 6

Assessment of age-related neurobehavioral phenotypes via a modified SHIRPA test battery.

Parameter	young+AL (n = 16)	young+EOD (n = 16)	old+AL (n = 23)	old+EOD (n = 23)
locomotor activity	23.25 ± 1.33	27.19 ± 1.15	15.26 ± 0.90	18.35 ± 0.94
tremor	all absent	all absent	22 absent, 1 present	21 absent, 2 present
defecation	9 present, 7 absent	8 present, 8 absent	6 present, 17 absent	11 present, 12 absent
transfer arousal	12 brief freeze, 4 immediate movement	16 of 16 immediate movement	21 brief freeze, 2 immediate movement	17 brief freeze, 6 immediate movement
gait	all fluid	all fluid	20 fluid, 3 abnormal	20 fluid, 3 abnormal
tail elevation	15 horizontal, 1 elevated	all horizontal	19 horizontal, 4 elevated	16 horizontal, 7 elevated
clickbox startle response	all preyer reflex	all preyer reflex	1 preyer reflex, 22 no reaction	4 preyer reflex, 19 no reaction
touch escape	all response to touch	all response to touch	all response to touch	all response to touch
trunk curl	all absent	all absent	all absent	all absent
limb gasping	all absent	all absent	all absent	all absent
pinna reflex	all present	all present	all present	all present
contact righting reflex	all present	all present	all present	all present
urination	5 present, 11 absent	9 present, 7 absent	3 present, 20 absent	5 present, 18 absent
evidence of biting	13 no aggression, 3 aggressive	14 no aggression, 2 aggressive	23 of 23 no aggression	22 no aggression, 1 aggressive
vocalisation	15 no, 1 yes	13 no, 3 yes	23 of 23 no	23 of 23 no
unexpected behavior	all none	all none	all none	all none
head bobbing	all absent	all absent	all absent	all absent

Supplementary Table 7

Open-field-based assessment of exploratory activity in a novel environment.

Parameter	young+AL (n = 16)	young+EOD (n = 16)	old+AL (n = 23)	old+EOD (n = 23)	effect of age (p-value)	effect of diet (p-value)	interaction (p-value)
total distance moved (m)	217.87 ± 5.13	234.86 ± 7.98	158.73 ± 9.80	199.97 ± 10.55	**<0.0001**	**0.0031**	0.2069
number of rears	165.13 ± 4.26	189.81 ± 5.89	106.43 ± 6.30	133.17 ± 5.51	**<0.0001**	**<0.0001**	0.8618
moved distance in center (%)	19.86 ± 1.06	22.15 ± 0.81	20.57 ± 1.05	26.78 ± 1.38	**0.0270**	**0.0006**	0.1023
time in center (%)	14.26 ± 0.84	16.61 ± 0.89	13.72 ± 1.14	18.54 ± 1.42	0.5670	**0.0042**	0.3102
total resting time (s)	145.01 ± 10.27	169.31 ± 9.31	76.81 ± 7.37	107.04 ± 6.39	**<0.0001**	**0.0014**	0.7194
average speed (cm/s)	20.69 ± 0.55	22.81 ± 0.76	14.19 ± 0.79	18.33 ± 1.00	**<0.0001**	**0.0008**	0.2611
moved distance in periphery (m)	174.48 ± 4.35	182.77 ± 6.45	126.03 ± 7.90	144.67 ± 6.41	**<0.0001**	0.0555	0.4574
resting time in periphery (s)	123.04 ± 7.97	142.61 ± 7.11	66.93 ± 6.06	87.47 ± 5.54	**<0.0001**	**0.0034**	0.9419
permanence in periphery (s)	1028.83 ± 10.05	1000.76 ± 10.72	1035.28 ± 13.72	977.46 ± 17.05	0.5636	**0.0041**	0.3087
speed in periphery (cm/s)	19.34 ± 0.56	21.36 ± 0.77	13.13 ± 0.87	16.48 ± 0.89	**<0.0001**	**0.0021**	0.4347
moved distance in center (m)	43.39 ± 2.62	52.08 ± 2.52	32.69 ± 2.58	55.29 ± 4.93	0.3108	**<0.0001**	0.0623
resting time in center (s)	21.98 ± 2.98	26.71 ± 3.13	9.89 ± 2.15	19.59 ± 2.17	**0.0004**	**0.0064**	0.3361
permanence in center (s)	171.18 ± 10.05	199.24 ± 10.72	164.72 ± 13.72	222.55 ± 17.06	0.5634	**0.0041**	0.3086
speed in center (cm/s)	29.03 ± 0.94	30.36 ± 0.81	21.71 ± 1.24	27.38 ± 1.26	**<0.0001**	**0.0042**	0.0705
latency to enter the center (s)	13.35 ± 3.47	10.15 ± 1.95	56.80 ± 14.39	54.77 ± 25.10	**0.0140**	0.8816	0.9734
number of center entries	214.31 ± 12.26	251.13 ± 11.34	162.83 ± 12.45	239.13 ± 20.92	0.0540	**0.0008**	0.2270

Supplementary Table 8

Assessment of aging-associated changes in optical density of the lens using Scheimpflug imaging.

Parameter	young+AL (n = 7)	young+EOD (n = 8)	old+AL (n = 7)	old+EOD (n = 6)	effect of age (p-value)	effect of diet (p-value)	interaction (p-value)
description of lens/cornea	all normal	all normal	all normal	all normal			
minimal density of left lens (%)	3.96 ± 0.16	3.95 ± 0.09	5.03 ± 0.22	4.10 ± 0.14	**0.0008**	**0.0069**	**0.0077**
maximal density of left lens (%)	7.26 ± 0.32	7.48 ± 0.22	8.67 ± 0.35	8.57 ± 0.95	**0.0203**	0.9075	0.7542
mean density of left lens (%)	5.03 ± 0.10	5.26 ± 0.20	6.43 ± 0.19	5.90 ± 0.23	**<0.0001**	0.4305	0.0513
minimal density of right lens (%)	4.41 ± 0.17	4.45 ± 0.21	4.47 ± 0.15	4.50 ± 0.23	0.7832	0.8688	0.9854
maximal density of right lens (%)	8.19 ± 0.37	7.86 ± 0.15	10.26 ± 0.58	10.08 ± 1.17	**0.0018**	0.6877	0.9037
mean density of right lens (%)	5.57 ± 0.11	5.79 ± 0.11	6.87 ± 0.18	6.87 ± 0.15	**<0.0001**	0.4488	0.4289

Supplementary Table 9

Assessment of the posterior part of the eye by optical coherence tomography.

Parameter	young+AL (n = 16)	young+EOD (n = 16)	old+AL (n = 21)	old+EOD (n = 22)	effect of age (p-value)	effect of diet (p-value)	interaction (p-value)
description of fundus	16 of 16 normal	14 normal, 2 abnormal	20 normal, 1 abnormal	21 normal, 1 abnormal			
pigmentation left fundus	all normal	all normal	all normal	all normal			
pigmentation right fundus	all normal	all normal	all normal	all normal			
optic disc left fundus	all normal	all normal	all normal	all normal			
optic disc right fundus	all normal	all normal	all normal	all normal			
number of vessels left fundus	9.44 ± 0.43	10.06 ± 0.31	9.55 ± 0.28	9.68 ± 0.38	0.7107	0.2968	0.4956
number of vessels right fundus	9.87 ± 0.46	10.25 ± 0.35	9.80 ± 0.30	10.45 ± 0.35	0.8532	0.1667	0.7161
retina left eye	all present	all present	all present	all present			
retina right eye	all present	all present	all present	all present			
retinal thickness left eye (µm)	251.00 ± 1.23	254.00 ± 1.51	252.65 ± 1.32	255.18 ± 1.34	0.3099	**0.0496**	0.8662
retinal thickness right eye (µm)	252.50 ± 1.38	253.06 ± 1.69	252.30 ± 1.24	252.45 ± 1.25	0.7733	0.7982	0.8843

Supplementary Table 10

Echocardiography-based assessment of cardiac dimensional and functional measures.

Parameter	young+AL (n = 10)	young+EOD (n = 9)	old+AL (n = 11)	old+EOD (n = 11)	effect of age (p-value)	effect of diet (p-value)	interaction (p-value)
heart rate (bpm)	537.091 ± 53.010	610.692 ± 26.244	553.858 ± 47.301	597.771 ± 33.289	0.9639	0.1720	0.7270
respiration rate (1/min)	181.180 ± 10.725	198.137 ± 16.087	191.732 ± 10.414	182.012 ± 13.037	0.8259	0.7752	0.2959
interventricular septum in diastole (mm)	0.562 ± 0.004	0.548 ± 0.005	0.576 ± 0.004	0.565 ± 0.006	**0.0030**	**0.0116**	0.8076
interventricular septum in systole (mm)	0.556 ± 0.004	0.559 ± 0.005	0.575 ± 0.006	0.570 ± 0.006	**0.0093**	0.8790	0.4959
left ventricular internal dimension in diastole (mm)	2.674 ± 0.152	2.268 ± 0.065	2.997 ± 0.161	2.738 ± 0.123	**0.0057**	**0.0187**	0.5897
left ventricular internal dimension in systole (mm)	1.400 ± 0.136	1.023 ± 0.082	1.645 ± 0.178	1.238 ± 0.087	0.0876	**0.0049**	0.9103
left ventricular posterior wall width in diastole (mm)	0.543 ± 0.006	0.539 ± 0.006	0.553 ± 0.002	0.553 ± 0.004	**0.0168**	0.6647	0.6647
left ventricular posterior wall in systole (mm)	0.541 ± 0.003	0.526 ± 0.006	0.545 ± 0.004	0.540 ± 0.005	0.0583	**0.0364**	0.2441
fractional shortening (%)	50.212 ± 2.926	56.384 ± 2.816	47.722 ± 3.610	57.423 ± 2.309	0.8093	**0.0116**	0.5584
left ventricular mass corrected (mg)	32.953 ± 2.733	24.081 ± 1.276	39.865 ± 4.054	33.411 ± 2.481	**0.0091**	**0.0134**	0.6841
ejection fraction (%)	81.952 ± 2.882	87.694 ± 2.091	78.477 ± 3.582	88.055 ± 1.584	0.5679	**0.0074**	0.4822
stroke volume (µl)	22.438 ± 2.523	15.444 ± 1.002	27.685 ± 2.847	25.274 ± 2.627	**0.0042**	0.0646	0.3595
cardiac output (ml/min)	11.529 ± 1.527	9.400 ± 0.716	14.767 ± 1.645	14.457 ± 1.106	**0.0038**	0.3695	0.5025

Supplementary Table 11

Electrocardiography-based assessment of electrical properties of the heart.

Parameter	young+AL (n = 10)	young+EOD (n = 9)	old+AL (n = 11)	old+EOD (n = 11)	effect of age (p-value)	effect of diet (p-value)	interaction (p-value)
heart rate (bpm)	749.50 ± 10.54	738.89 ± 8.37	737.64 ± 8.20	665.36 ± 14.35	**0.0004**	**0.0005**	**0.0077**
heart rate variability (bpm)	26.02 ± 15.08	36.60 ± 22.33	27.88 ± 13.83	22.21 ± 14.32	0.7028	0.8811	0.6209
signal intensity (%)	3.35 ± 1.92	4.80 ± 2.86	3.64 ± 1.77	3.25 ± 2.03	0.7705	0.8059	0.6684
RR interval (ms)	80.62 ± 0.99	81.79 ± 0.87	81.65 ± 0.79	90.87 ± 1.95	**0.0004**	**0.0003**	**0.0036**
PQ interval (ms)	20.72 ± 1.00	19.54 ± 1.23	16.18 ± 1.52	16.12 ± 1.38	**0.0048**	0.6435	0.6779
PR interval (ms)	26.45 ± 1.26	25.74 ± 1.32	22.45 ± 1.47	23.24 ± 1.31	**0.0216**	0.9751	0.5848
QRS interval (ms)	9.82 ± 0.28	10.08 ± 0.16	10.31 ± 0.21	11.80 ± 0.59	**0.0051**	**0.0239**	0.1050
QT interval (ms)	41.26 ± 0.79	39.26 ± 0.95	41.63 ± 0.74	46.45 ± 1.24	**0.0004**	0.1527	**0.0011**
ST interval (ms)	31.93 ± 0.81	29.69 ± 0.85	31.83 ± 0.74	35.11 ± 0.96	**0.0034**	0.5443	**0.0025**
QTc interval (ms)	45.99 ± 0.63	43.50 ± 0.93	46.11 ± 0.73	48.72 ± 0.99	**0.0030**	0.9438	**0.0044**
QT dispersion (ms)	22.30 ± 1.75	23.06 ± 1.52	18.36 ± 0.98	20.59 ± 2.28	0.0714	0.3926	0.6720
QTc dispersion (ms)	24.90 ± 2.00	27.84 ± 2.62	22.15 ± 1.38	22.09 ± 1.82	**0.0350**	0.4619	0.4453
SR amplitude (mV)	0.93 ± 0.11	0.86 ± 0.11	0.77 ± 0.12	0.78 ± 0.14	0.3286	0.7660	0.7438
R amplitude (mV)	0.68 ± 0.08	0.67 ± 0.08	0.61 ± 0.09	0.63 ± 0.10	0.5917	0.9841	0.8504
rMSSD (ms)	2.85 ± 1.64	3.84 ± 2.25	3.02 ± 1.33	2.52 ± 1.10	0.7149	0.8775	0.6391
pNN50 (%)	10.56 ± 9.95	1.08 ± 0.73	4.44 ± 3.39	1.23 ± 0.84	0.5722	0.2327	0.5528

rMSSD = root mean square of the successive differences of neighboring RR intervals, pNN50 = percentage of adjacent RR intervals with differences larger than 50 ms

Supplementary Table 12

Assessment of bone structure via micro-CT.

Parameter	young+AL (n = 16)	young+EOD (n = 9)	old+AL (n = 17)	old+EOD (n = 9)	effect of age (p-value)	effect of diet (p-value)	interaction (p-value)
total tissue area (mm^2)	0.730 ± 0.011	0.738 ± 0.019	0.757 ± 0.014	0.754 ± 0.016	0.1753	0.8625	0.7165
bone area (mm^2)	0.615 ± 0.009	0.616 ± 0.017	0.625 ± 0.014	0.625 ± 0.015	0.4948	0.9889	0.9657
marrow area (mm^2)	0.115 ± 0.002	0.122 ± 0.003	0.131 ± 0.001	0.129 ± 0.002	**<0.0001**	0.2072	**0.0130**
polar moment of inertia (mm^4)	0.109 ± 0.004	0.126 ± 0.007	0.154 ± 0.004	0.150 ± 0.006	**<0.0001**	0.2481	0.0525
cortical thickness (mm)	0.219 ± 0.003	0.199 ± 0.007	0.178 ± 0.004	0.179 ± 0.004	**<0.0001**	**0.0397**	**0.0304**

Supplementary Table 13

Analysis of metabolic parameters using indirect calorimetry.

Parameter	young+AL (n = 16)	young+EOD (n = 16)	old+AL (n = 16)	old+EOD (n = 16)	effect of age (p-value)	effect of diet (p-value)	interaction (p-value)
O_2 consumption (ml/h)	79.71 ± 1.57	75.52 ± 2.01	90.79 ± 2.07	80.24 ± 1.62	**<0.0001**	**0.0002**	0.0883
O_2 consumption/body weight (ml/h per g body weight)	2.76 ± 0.06	3.07 ± 0.09	2.25 ± 0.06	2.58 ± 0.05	**<0.0001**	**<0.0001**	0.9423
CO_2 production (ml/h)	70.50 ± 1.77	66.57 ± 2.04	74.46 ± 1.61	69.34 ± 1.59	0.0609	**0.0126**	0.7373
CO_2 production/body weight (ml/h per g body weight)	2.44 ± 0.07	2.71 ± 0.09	1.86 ± 0.06	2.23 ± 0.05	**<0.0001**	**<0.0001**	0.4368
mean respiratory exchange rate	0.88 ± 0.01	0.87 ± 0.01	0.82 ± 0.01	0.86 ± 0.01	**0.0002**	0.0799	**0.0076**
respiratory exchange rate (feasting day)	0.86 ± 0.01	0.93 ± 0.01	0.81 ± 0.01	0.89 ± 0.01	**<0.0001**	**<0.0001**	0.6842
respiratory exchange rate (fasting day)	0.89 ± 0.01	0.78 ± 0.01	0.83 ± 0.02	0.81 ± 0.01	0.2212	**<0.0001**	**0.0003**
heat production (kcal/h)	0.39 ± 0.01	0.37 ± 0.01	0.44 ± 0.01	0.39 ± 0.01	**0.0003**	**0.0004**	0.1510
total movement x-axis per minute	27.84 ± 1.28	35.68 ± 2.51	35.15 ± 2.79	44.12 ± 4.83	**0.0145**	**0.0093**	0.8570
ambulatory movement x-axis per minute	19.19 ± 0.83	26.13 ± 1.96	24.89 ± 3.13	33.05 ± 3.99	**0.0143**	**0.0037**	0.8090
fine movement x-axis per minute	8.65 ± 0.56	9.55 ± 0.69	10.26 ± 0.81	11.07 ± 0.87	**0.0400**	0.2557	0.9557
total movement y-axis per minute	34.92 ± 3.07	40.19 ± 2.18	32.07 ± 2.08	39.24 ± 3.79	0.5104	**0.0339**	0.7423
ambulatory movement y-axis per minute	22.14 ± 1.43	27.62 ± 1.62	20.88 ± 1.65	26.57 ± 2.96	0.5667	**0.0072**	0.9578
fine movement y-axis per minute	12.78 ± 1.68	12.57 ± 0.61	11.19 ± 0.47	12.67 ± 0.88	0.4719	0.5364	0.4154
rearing movement z-axis per minute	6.64 ± 0.87	8.41 ± 1.15	11.66 ± 1.83	10.40 ± 0.96	**0.0072**	0.8401	0.2339
moved distance (cm/min)	170.27 ± 20.67	205.90 ± 22.53	173.45 ± 12.64	209.00 ± 20.52	0.8723	0.0722	0.9983
speed (cm/s)	2.83 ± 0.34	3.42 ± 0.37	2.87 ± 0.21	3.48 ± 0.34	0.8746	0.0702	0.9793
mean food intake (g/day)	2.55 ± 0.17	2.08 ± 0.16	1.55 ± 0.21	1.51 ± 0.22	**0.0001**	0.1898	0.2589
food intake/body weight (g/day per g body weight)	0.09 ± 0.01	0.08 ± 0.01	0.04 ± 0.01	0.05 ± 0.01	**<0.0001**	0.7335	0.3214
mean water consumption (ml/day)	3.32 ± 0.16	4.08 ± 0.43	2.22 ± 0.28	3.46 ± 0.25	**0.0068**	**0.0013**	0.0937
water consumption/body weight (ml/day per g body weight)	0.12 ± 0.01	0.15 ± 0.01	0.06 ± 0.01	0.11 ± 0.01	**<0.0001**	**<0.0001**	0.2821
water consumption (ml/day) (feeding day)	3.21 ± 0.21	5.84 ± 0.49	2.02 ± 0.35	5.22 ± 0.55	**0.0348**	**<0.0001**	0.5019
water consumption (ml/day) (fasting day)	3.43 ± 0.17	1.63 ± 0.22	2.42 ± 0.32	1.71 ± 0.33	0.0889	**<0.0001**	**0.0466**

Supplementary Table 14

Basic hematological analyses.

Parameter	young+AL (n = 16)	young+EOD (n = 16)	old+AL (n = 20)	old+EOD (n = 22)	effect of age (p-value)	effect of diet (p-value)	interaction (p-value)
white blood cell counts ($10^3/mm^3$)	12.30 ± 0.55	8.86 ± 0.52	14.69 ± 1.57	9.14 ± 0.83	0.2091	**<0.0001**	0.3195
red blood cell counts ($10^6/mm^3$)	11.53 ± 0.20	11.42 ± 0.29	9.53 ± 0.36	9.88 ± 0.13	**<0.0001**	0.6349	0.3859
platelet counts ($10^6/mm^3$)	1.17 ± 0.05	1.22 ± 0.03	2.31 ± 0.12	2.34 ± 0.11	**<0.0001**	0.7129	0.9619
hemoglobin (g/dl)	17.47 ± 0.28	18.06 ± 0.43	14.24 ± 0.42	15.50 ± 0.21	**<0.0001**	**0.0097**	0.3340
hematocrit (%)	53.92 ± 0.87	56.07 ± 1.39	44.76 ± 1.23	48.63 ± 0.63	**<0.0001**	**0.0054**	0.4129
mean corpuscular volume (fl)	46.88 ± 0.15	49.25 ± 0.14	47.75 ± 1.36	49.23 ± 0.30	0.5826	**0.0149**	0.5627
mean corpuscular hemoglobin content (pg)	15.16 ± 0.08	15.83 ± 0.10	15.13 ± 0.30	15.69 ± 0.13	0.6639	**0.0020**	0.7910
mean corpuscular hemoglobin concentration (g/dl)	32.40 ± 0.16	32.23 ± 0.20	31.77 ± 0.29	31.88 ± 0.21	**0.0368**	0.8923	0.5358
red blood cell distribution width (%)	14.31 ± 0.18	13.49 ± 0.13	15.50 ± 0.49	13.67 ± 0.15	**0.0253**	**<0.0001**	0.0959
platelet volume (fl)	6.07 ± 0.03	6.02 ± 0.02	6.41 ± 0.08	6.25 ± 0.03	**<0.0001**	**0.0450**	0.2994
platelet distribution width (%)	5.32 ± 0.04	5.23 ± 0.03	5.76 ± 0.12	5.55 ± 0.04	**<0.0001**	**0.0440**	0.4476
platelet large cell ratio (%)	1.83 ± 0.11	1.67 ± 0.10	3.20 ± 0.40	2.43 ± 0.11	**<0.0001**	0.0585	0.2084
plateletcrit (%)	0.71 ± 0.03	0.73 ± 0.02	1.47 ± 0.08	1.46 ± 0.07	**<0.0001**	0.9500	0.8447

Supplementary Table 15

FACS-based leukocyte profiling in the peripheral blood.

Parameter	young+AL (n = 16)	young+EOD (n = 16)	old+AL (n = 20)	old+EOD (n = 22)	effect of age (p-value)	effect of diet (p-value)	interaction (p-value)
T cells (% of leukocytes)	16.27 ± 0.48	19.71 ± 0.43	11.53 ± 0.81	15.37 ± 0.81	**<0.0001**	**<0.0001**	0.7860
$Ly6C^{+}$ T cells (% of T cells)	22.13 ± 0.65	24.12 ± 0.63	44.53 ± 2.07	42.05 ± 1.98	**<0.0001**	0.8848	0.1910
B1 cells (% of leukocytes)	1.07 ± 0.17	0.94 ± 0.13	0.69 ± 0.08	0.90 ± 0.13	0.1107	0.7689	0.1883
B2 cells (% of leukocytes)	61.94 ± 1.13	55.53 ± 1.13	48.29 ± 3.77	48.97 ± 3.46	**0.0013**	0.3443	0.2433
$CD11b^{+}$ B cells (% of B cells)	1.45 ± 0.15	1.20 ± 0.08	3.43 ± 0.46	3.00 ± 0.43	**<0.0001**	0.3635	0.8008
$Ly6C^{+}$ B cells (% of B cells)	4.59 ± 0.28	4.09 ± 0.37	7.96 ± 1.13	5.64 ± 0.62	**0.0017**	0.0641	0.2304
NK cells (% of leukocytes)	2.12 ± 0.11	2.04 ± 0.08	1.12 ± 0.08	0.96 ± 0.07	**<0.0001**	0.1390	0.6242
$CD11b^{+}$ NK cells (% of NK cells)	87.88 ± 0.44	84.83 ± 0.56	81.16 ± 0.86	76.75 ± 1.26	**<0.0001**	**0.0002**	0.4783
$CD11c^{+}$ NK cells (% of NK cells)	43.94 ± 0.78	40.38 ± 1.32	50.47 ± 1.58	40.77 ± 1.31	**0.0123**	**<0.0001**	**0.0258**
$Ly6C^{+}$ NK cells (% of NK cells)	52.01 ± 0.86	57.06 ± 1.15	49.72 ± 1.77	57.85 ± 1.21	0.5815	**<0.0001**	0.2617
NKT cells (% of leukocytes)	0.46 ± 0.05	0.40 ± 0.04	0.18 ± 0.02	0.40 ± 0.06	**0.0050**	0.1141	**0.0048**
granulocytes (% of leukocytes)	9.75 ± 0.44	9.22 ± 0.93	22.98 ± 3.19	20.46 ± 3.28	**<0.0001**	0.5704	0.7106
$Ly6C^{+}$ monocytes (% of leukocytes)	6.35 ± 0.50	9.41 ± 0.54	11.39 ± 1.01	9.73 ± 0.77	**0.0011**	0.3727	**0.0038**
$Ly6C^{-}$ monocytes (% of leukocytes)	1.74 ± 0.11	2.36 ± 0.15	3.09 ± 0.24	2.59 ± 0.30	**0.0014**	0.8061	**0.0215**
$Ly6C^{+}/CD11C^{+}$ monocytes (% of monocytes)	0.61 ± 0.05	0.55 ± 0.04	0.60 ± 0.05	0.56 ± 0.05	0.9524	0.3554	0.8508
$Ly6C^{+}/CD11C^{-}$ monocytes (% of monocytes)	46.04 ± 1.98	44.60 ± 2.91	52.58 ± 1.77	51.24 ± 2.82	**0.0100**	0.5774	0.9832
$Ly6C^{(+)}/CD11C^{+}$ monocytes (% of monocytes)	6.26 ± 0.24	4.82 ± 0.30	5.22 ± 0.14	5.40 ± 0.29	0.3622	**0.0158**	**0.0020**
$Ly6C^{(+)}/CD11C^{-}$ monocytes (% of monocytes)	24.96 ± 2.14	29.69 ± 2.92	19.35 ± 1.50	21.49 ± 2.02	**0.0020**	0.1147	0.5513
$Ly6C^{-}/CD11C^{+}$ monocytes (% of monocytes)	12.36 ± 0.54	11.74 ± 0.69	11.89 ± 0.44	12.00 ± 0.77	0.8752	0.6925	0.5770
$Ly6C^{-}/CD11C^{-}$ monocytes (% of monocytes)	9.76 ± 0.71	8.61 ± 0.44	10.37 ± 0.83	9.31 ± 0.82	0.3954	0.1526	0.9523
$CD45^{+}$ non specific cells (% of leukocytes)	0.29 ± 0.03	0.37 ± 0.06	0.72 ± 0.09	0.62 ± 0.09	**<0.0001**	0.9420	0.2319

$Ly6C^{+}$ = Ly6C high, $Ly6C^{(+)}$ = Ly6C medium, $Ly6C^{-}$ = Ly6C negative.

Supplementary Table 16

Clinical chemistry measures in the fed state.

Parameter	young+AL (n = 16)	young+EOD (n = 16)	old+AL (n = 20)	old+EOD (n = 22)	effect of age (p-value)	effect of diet (p-value)	interaction (p-value)
Na (mmol/l)	154.81 ± 0.44	155.19 ± 0.34	156.20 ± 0.66	155.91 ± 0.32	**0.0295**	0.9296	0.4851
K (mmol/l)	4.14 ± 0.06	4.13 ± 0.08	4.42 ± 0.09	4.70 ± 0.09	**<0.0001**	0.1441	0.0949
Ca (mmol/l)	2.34 ± 0.01	2.35 ± 0.01	2.39 ± 0.02	2.32 ± 0.02	0.7043	**0.0389**	**0.0221**
Cl (mmol/l)	112.10 ± 0.43	112.79 ± 0.35	112.09 ± 0.60	112.86 ± 0.38	0.9571	0.1201	0.9316
inorganic phosphate (mmol/l)	1.71 ± 0.08	1.47 ± 0.06	1.76 ± 0.04	1.62 ± 0.05	0.1000	**0.0016**	0.3776
total protein (g/l)	49.96 ± 0.46	49.19 ± 0.46	51.41 ± 0.73	48.98 ± 0.59	0.3075	**0.0098**	0.1696
urea (mmol/l)	11.20 ± 0.43	11.74 ± 0.49	13.93 ± 0.58	12.22 ± 0.43	**0.0020**	0.2442	**0.0276**
cholesterol (mmol/l)	2.41 ± 0.12	2.60 ± 0.04	2.47 ± 0.05	2.34 ± 0.09	0.2208	0.7079	**0.0420**
triglycerides (mmol/l)	1.52 ± 0.12	1.41 ± 0.13	1.67 ± 0.11	1.23 ± 0.09	0.9142	**0.0181**	0.1513
GPT activity (U/l)	46.06 ± 4.06	40.87 ± 4.28	57.50 ± 9.31	53.00 ± 8.35	0.1313	0.5320	0.9642
GOT activity (U/l)	62.63 ± 5.81	54.27 ± 2.14	68.37 ± 4.38	63.29 ± 4.43	0.1130	0.1483	0.7227
AP activity (U/l)	82.88 ± 3.26	81.31 ± 2.26	95.55 ± 5.70	88.27 ± 4.29	**0.0290**	0.3189	0.5184
glucose (mmol/l)	13.57 ± 0.45	12.63 ± 0.55	11.37 ± 0.32	11.13 ± 0.43	**<0.0001**	0.1864	0.4299
lactate (mmol/l)	11.58 ± 0.33	12.18 ± 0.22	12.16 ± 0.28	12.35 ± 0.28	0.1966	0.1750	0.4910
Fe (µmol/l)	20.03 ± 0.49	41.95 ± 2.95	23.57 ± 1.60	33.06 ± 1.30	0.1312	**<0.0001**	**0.0007**
UIBC (µmol/l)	39.69 ± 1.25	16.81 ± 3.52	45.73 ± 2.40	33.72 ± 3.46	**0.0003**	**<0.0001**	0.0763
LDH activity (U/l)	164.19 ± 22.75	123.25 ± 12.94	225.15 ± 13.13	187.33 ± 17.83	**0.0006**	**0.0269**	0.9289
albumin (g/l)	27.97 ± 0.58	28.49 ± 0.22	27.25 ± 0.41	26.99 ± 0.54	**0.0237**	0.7828	0.4173
α-amylase activity (U/l)	800.09 ± 27.21	764.30 ± 12.42	895.18 ± 54.41	793.15 ± 21.66	0.0792	0.0516	0.3444
fructosamine (µmol/l)	321.19 ± 8.57	320.81 ± 8.49	301.16 ± 9.08	306.36 ± 8.55	0.0573	0.7873	0.7553

AP = alkaline phosphatase, GOT = aspartate-aminotransferase (ASAT), GPT = alanine-aminotransferase (ALAT), LDH = lactate-dehydrogenase, UIBC = unsaturated iron binding capacity.

Supplementary Table 17

Clinical chemistry measures after a 6-hour fasting period.

Parameter	young+AL (n = 16)	young+EOD (n = 16)	old+AL (n = 23)	old+EOD (n = 23)	effect of age (p-value)	effect of diet (p-value)	interaction (p-value)
fasting cholesterol (mmol/l)	2.463 ± 0.080	2.374 ± 0.060	2.667 ± 0.051	2.260 ± 0.038	0.4752	**0.0002**	**0.0136**
fasting triglycerides (mmol/l)	0.774 ± 0.044	0.633 ± 0.019	0.758 ± 0.055	0.634 ± 0.030	0.8696	**0.0038**	0.8422
fasting glucose (mmol/l)	15.356 ± 0.432	14.356 ± 0.306	15.483 ± 0.400	14.170 ± 0.417	0.9456	**0.0098**	0.7201
fasting HDL-cholesterol (mmol/l)	1.732 ± 0.057	1.583 ± 0.040	1.863 ± 0.038	1.491 ± 0.031	0.6656	**<0.0001**	**0.0173**
fasting non HDL-cholesterol (mmol/l)	0.731 ± 0.024	0.791 ± 0.023	0.804 ± 0.020	0.769 ± 0.015	0.2606	0.5659	**0.0354**
fasting glycerol (mmol/l)	0.102 ± 0.004	0.124 ± 0.004	0.129 ± 0.006	0.118 ± 0.004	0.0525	0.2711	**0.0029**
fasting NEFA (mmol/l)	0.806 ± 0.054	0.655 ± 0.016	0.661 ± 0.027	0.648 ± 0.028	**0.0428**	**0.0295**	0.0644

HDL = high density lipoprotein, NEFA = non-esterified fatty acids.
